# Reply to Chen et al.: Parametric methods for cluster inference perform worse for two‐sided *t*‐tests

**DOI:** 10.1002/hbm.24465

**Published:** 2018-12-07

**Authors:** Anders Eklund, Hans Knutsson, Thomas E. Nichols

**Affiliations:** ^1^ Division of Medical Informatics, Department of Biomedical Engineering Linköping University Linköping Sweden; ^2^ Division of Statistics and Machine Learning, Department of Computer and Information Science Linköping University Linköping Sweden; ^3^ Center for Medical Image Science and Visualization (CMIV) Linköping University Linköping Sweden; ^4^ Big Data Institute University of Oxford Oxford United Kingdom; ^5^ Wellcome Trust Centre for Integrative Neuroimaging (WIN‐FMRIB) University of Oxford Oxford United Kingdom; ^6^ Department of Statistics University of Warwick Coventry United Kingdom

**Keywords:** cluster inference, false positives, fMRI, one‐sided, permutation, two‐sided

## Abstract

One‐sided *t*‐tests are commonly used in the neuroimaging field, but two‐sided tests should be the default unless a researcher has a strong reason for using a one‐sided test. Here we extend our previous work on cluster false positive rates, which used one‐sided tests, to two‐sided tests. Briefly, we found that parametric methods perform worse for two‐sided *t*‐tests, and that nonparametric methods perform equally well for one‐sided and two‐sided tests.

## 1 INTRODUCTION

Chen et al. (2018) discuss an important topic which is often neglected in the neuroimaging field, the use of one‐sided or two‐sided tests and the lack of multiple comparison correction for two one‐sided tests. As mentioned in their article, in our work on massive empirical evaluation of task fMRI inference methods with resting state fMRI (Eklund, Nichols, & Knutsson, 2016) we used one‐sided tests (familywise error rate *α*_FWE_ = 0.05). We made this choice for two reasons. The first reason was simply that for analyses of randomly created groups of healthy controls, it should make no difference if one uses a one‐sided or a two‐sided test. The second reason was more practical. FSL and SPM both run one‐sided tests by default, and we wished to reflect the typical (if ill‐advised) practices of the community. Furthermore, to perform a two‐sided permutation test (Winkler, Ridgway, Webster, Smith, & Nichols, 2014), it would be necessary to run two permutation tests per group analysis (which would double the processing time), since normally only the maximum test value over the brain (or the largest cluster) is saved for every permutation (to form the maximum null distribution).

## 2 METHODS

To investigate if performing a two‐sided test (as implemented by two tests at *α*_FWE_ = 0.025) lead to different false positive rates compared with a single one‐sided test (at *α*_FWE_ = 0.05), we performed new group analyses for a subset of all the parameter settings used in our previous work (Eklund et al., 2016; Eklund, Knutsson, & Nichols, 2018). Specifically, we only performed two‐sample *t*‐tests for the Beijing data (Biswal, Mennes, Zuo, & Milham, 2010), using 40 subjects (i.e., 20 subjects per group) and a cluster defining threshold of *p* = .001. All group analyses were performed for 4, 6, 8, and 10 mm FWHM of smoothing. See our recent work (Eklund et al., 2018) for a description of the six designs (B1, B2, E1, E2, E3, and E4) applied to every subject in the first level analysis.

For FSL, group analyses were only performed using FSL OLS, and not using FLAME1 (which is the default option); FLAME1 leads to conservative results if resting state fMRI data is used, while null task fMRI analyses (control–control) with FLAME1 gives FWE rates comparable to FSL OLS (Eklund et al., 2016). For AFNI, we used the new autocorrelation function (ACF) option in 3dClustSim (Cox, Chen, Glen, Reynolds, & Taylor, 2017), which uses a long‐tail spatial ACF instead of a Gaussian one. It should be noted that AFNI provides another function for cluster thresholding, equitable thresholding and clustering (ETAC) (Cox, 2018), which may perform better than the long‐tail ACF function used here, but we used the ACF approach to be able to compare the two‐sided results to our recent work (Eklund et al., 2018). Contrary to Chen et al. (2018), we did not change the cluster defining threshold to *p* = .0005 when performing two one‐sided tests (for SPM, FSL, or AFNI), as this represents yet another change in the inference configuration that we rather leave fixed to facilitate the comparison of these results to previous one‐sided findings.

## 3 RESULTS

**Figure 1:**
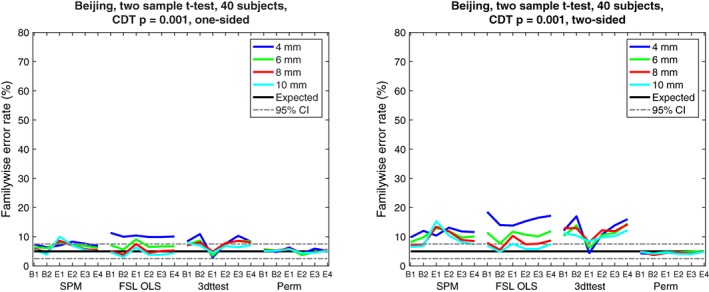
A comparison of empirical familywise error rates for one‐sided (left) and two‐sided (right) tests, for a cluster defining threshold of *p* = .001. Designs B1 and B2 represent two block based activity paradigms, while E1, E2, E3, and E4 represent event related paradigms. Design E4 is randomized over subjects, while all other designs are the same for all subjects. The parametric methods perform worse for two one‐sided tests at *α*_FWE_ = 0.025, compared with a single one‐sided test at *α*_FWE_ = 0.05, while the permutation test produces nominal results in both cases [Color figure can be viewed at http://wileyonlinelibrary.com]

Figure 1 shows estimated familywise error rates for one‐sided and two‐sided tests, where both should exhibit a nominal 5% familywise false positive rate. The nonparametric permutation test produces similar results in both cases, while the parametric methods perform worse for two‐sided tests.

## 4 DISCUSSION

We have extended our original work on cluster false positive rates (Eklund et al., 2016, 2018) to two‐sided tests, showing that parametric methods perform worse for two‐sided tests. RFT *p*‐values depend on a number of approximations:

1. Joint normality over the image,
2. Sufficient smoothness for lattice images to behave like continuous processes,
3. Homogeneous smoothness (stationarity), so that the null distribution of cluster size does not vary over space,
4. Spatial dependence mostly local, that is, the spatial autocorrelation function is proportional to a Gaussian density, and
5. Sufficiently high cluster‐forming threshold so that the approximate distribution for cluster size is accurate.

On this last assumption, the control of FWE depends on the accuracy of the cluster size distribution in its tail. For example, it is of little consequence if the true cluster size FWE *p*‐value is .6 and RFT estimates it as .5; in contrast, two‐sided inference demands accuracy in the RFT approximation down to FWE 0.025, and then any inaccuracies are doubled as both positive and negative excursions are considered. In our findings, it appears that modest inaccuracies in the null cluster size distribution corresponding to FWE 0.05 (see Figure 1a, and general tendency to overestimate FWE) grow into larger inaccuracies when the more stringent FWE level 0.025 is used (the inference used twice for each result contributing to Figure 1b).

In contrast, the nonparametric permutation test for a two‐sample *t*‐test is only based on the assumption of exchangeability between subjects, and therefore performs equally well for two one‐sided tests at *α*_FWE_ = 0.025.
